# A Spectrofluorometric Method for Real‐Time Graft Assessment and Patient Monitoring

**DOI:** 10.1002/advs.202301537

**Published:** 2023-06-02

**Authors:** Florian Huwyler, Janina Eden, Jonas Binz, Leslie Cunningham, Richard X. Sousa Da Silva, Pierre‐Alain Clavien, Philipp Dutkowski, Mark W. Tibbitt, Max Hefti

**Affiliations:** ^1^ Macromolecular Engineering Lab, Department of Mechanical and Process Engineering ETH Zurich Zurich 8092 Switzerland; ^2^ Department of Surgery and Transplantation, Swiss Hepato‐Pancreato‐Biliary (HPB) and Transplant Center University Hospital Zurich Zurich 8091 Switzerland; ^3^ Wyss Zurich Translational Center ETH Zurich and University of Zurich Zurich 8092 Switzerland

**Keywords:** biomarkers, biomedical engineering, biosensors, liver transplantation, translational research

## Abstract

Biomarkers are powerful clinical diagnostics and predictors of patient outcome. However, robust measurements often require time and expensive laboratory equipment, which is insufficient to track rapid changes and limits direct use in the operating room. Here, this study presents a portable spectrophotometric device for continuous real‐time measurements of fluorescent and non‐fluorescent biomarkers at the point of care. This study measures the mitochondrial damage biomarker flavin mononucleotide (FMN) in 26 extended criteria human liver grafts undergoing hypothermic oxygenated perfusion to guide clinical graft assessment. Real‐time data identified seven organs unsuitable for transplant that are discarded. The remaining grafts are transplanted and FMN values correlated with post‐transplant indicators of liver function and patient recovery. Further, this study shows how this device can be used to monitor dialysis patients by measuring creatinine in real‐time. Our approach provides a simple method to monitor biomarkers directly within biological fluids to improve organ assessment, patient care, and biomarker discovery.

## Graphical abstract

Fluorescent assays are systematically developed to continuously measure biomarkers in real‐time and non‐invasively, enabling the clinical use of novel biomarkers. The approach is validated by measuring flavin mononucleotide in human liver grafts to predict transplant outcome. Real‐time biomarker data correlates with patient recovery after transplantation and constitutes a reliable indicator for safer transplantation of extended criteria donor grafts.

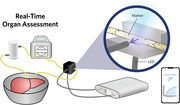

## 1 Introduction

Biomarkers are an integral part of clinical assessment strategies in modern medicine, but are usually only evaluated at discrete time points and often require elaborate measurements in a laboratory.^[^ ^1^, ^2^, ^3^, ^4^, ^5^ ^]^ This is sufficient for diseases that progress over months and years, such as prostate cancer, where prostate specific antigen measurements are used during routine screenings, separated by several months or even years.^[^ ^6^, ^7^, ^8^ ^]^ However, many diseases or injuries are highly dynamic with biomarkers changing in the time frame of minutes. For instance, patients suffering an ischemic stroke exhibit rapid changes in biomarkers, such as S100 calcium binding protein B, neuronspecific enolase, myelin basic protein, and glial fibrillary acidic protein, which can be used for diagnosis and prediction.^[^ ^9^ ^]^ In such situations, time lags introduced by laboratory measurements can have severe consequences and make it difficult to gather sufficient data to monitor injury progression, impacting the quality of patient care. Therefore, there is a clinical need to develop methods for biomarker measurements that enable simple use and continuous real‐time data collection directly in the clinics. This would make the use of biomarkers available to more clinicians, thereby improving patient care in clinical settings, and would provide more data to improve our understanding of rapidly progressing injury mechanisms.

Here, we introduce a method to translate biomarkers from their discovery to clinical practice. We formulated four key steps in the development of such biomarker assays: The discovery of a marker, the development of a fluorescent assay, the engineering of a calibrated spectrofluorometric device, and the validation with an independent analytical technique. By describing these steps based on two examples with clinical utility, we show the development of next generation diagnostic devices that continuously measure biomarkers in real‐time, directly within biological fluids, with high accuracy and without expensive equipment. A modular design facilitates integration of additional biomarkers, that can be measured simultaneously. Unlike previous methods that required discrete sampling, our portable devices are non‐invasive and do not require scientific staff to perform laboratory analysis. Clinicians further profit from continuous data sets, which offer more information on biomarker trends and can be inspected in real‐time, saving time when it matters the most. By developing a custom interface and an iOS App, clinicians can trace biomarkers of patients or organs right on their mobile phone, freeing them to perform other duties. We further show a calibration approach that helps harmonizing biomarker units among the communities, improving reproducibility, and comparability. Ultimately, we show the development of diagnostic tools that facilitate the use of novel biomarkers, thereby increasing clinical use and broadening the impact of biomarkers.

We demonstrate continuous real‐time measurements of two fluorescent markers, FMN and nicotinamide adenine dinucleotide (NADH), and one non‐fluorescent marker, creatinine. In the case of FMN, we illustrate the direct clinical impact of real‐time biomarker measurements for organ assessment prior to transplantation. We measured FMN concentrations during hypothermic oxygenated perfusion (HOPE) of 26 human liver grafts in real‐time with high precision. The data guided transplant decisions, discriminating between transplantable and non‐transplantable grafts. The evolution of FMN concentration was compared to post‐operative clinical data of 19 transplanted patients, demonstrating robust correlations between the biomarker values during perfusion and transplant outcome. Further, we measured creatinine concentrations in whole blood non‐invasively, demonstrating the ability of our method to quantify fluorescent and non‐fluorescent biomarkers in non‐transparent fluids. This approach could allow real‐time monitoring of creatinine clearance in dialysis patients, showing the efficacy of the renal replacement therapy. By allowing clinicians to use biomarkers on site and with real‐time data, we offer a simple method to leverage the potential of biomarkers, leading to better patient diagnosis and understanding of complex injuries.

## 2 Results

### 2.1 Method Development

Translating biomarkers to clinical practice requires four critical steps: marker discovery, development of a measurement assay, device engineering & calibration, and validation. Marker discovery requires the identification of one or more biomarkers whose levels correlate with a clinical diagnosis. Many recent biomarkers have been reported, but translating them requires robust methods that can be implemented in clinical practice. The most common techniques for clinical biomarker measurements are enzyme‐linked immunosorbent assay (ELISA) and liquid chromatography mass spectrometry (LC‐MS).^[^ ^1^, ^10^, ^11^, ^12^ ^]^ However, ELISA and LC‐MS require discrete samples to be taken from the biological fluid and, therefore, introduce the risk of contamination and do not allow for real‐time measurements. Many biomarker quantification techniques, including ELISA, rely on absorbance or fluorescence and are read on commercial spectrometers and plate readers. Spectrofluorometric measurements often introduce lab‐to‐lab variability owing to differences in optical components, which can make clinical standardization challenging. Nonetheless, simple optical measurements offer an opportunity to assess biomarkers continuously and in the clinic, provided that a robust and non‐invasive device exists.

**Figure 1:**
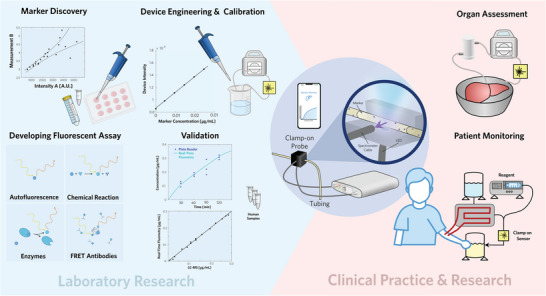
Translating biomarker measurements from laboratories to the operating room: Fundamental research and method development (left), include initial marker discovery, development of a fluorometric assay by using one of four approaches, device engineering, and calibration, and validation by using independent analytical techniques. The benefits for clinical practice and research (right) involve organ assessment and patient monitoring, either for improved patient care or to gather more data for clinical research. We developed a portable spectrofluorometric device for real‐time measurements of biomarkers (center). The spectrometer and light source are connected to tubing carrying perfusate or dialysate and data can be tracked on a mobile application or computer graphical interface.

We identified four potential mechanisms as to how the concentration of a biomarker could be measured via a spectrofluorometric device in real‐time within biological fluids **Figure** **1**. First, there are biomarkers that are colorimetric or autofluorescent wherein an optical or fluorescent signal is proportional to the marker concentration.^[^ ^13^ ^]^ Such biomarkers can be measured directly in a flow cell through, which the biological fluid or perfusate is circulated. Second, chemical reagents can be added to the biological fluid, driving a chemical reaction to make the biomarker or reaction products fluorescent. Third, fluorescent properties of marker complexes can also be induced by enzymes, analogously to ELISA. The fourth, and arguably most sophisticated method is fluorescence resonance energy transfer (FRET), where the biomarker binds soluble donor and acceptor fluorophores, bringing them near enough for non‐radiative FRET to occur. Here, absorbed energy from the donor is passed on to the acceptor and the acceptor radiatively loses this energy in form of fluorescence. FRET‐antibody solutions are commercially available for many proteins, including IL‐1β, IL‐6, IL‐11, and TNF‐α, and allow detection of markers when mixed with a sample solution.

In order to apply any of these approaches for real‐time measurement of biomarkers within complex biological fluids, a non‐invasive and robust method is required. Therefore, we engineered a portable spectrofluorometric device with a computer interface to analyze biological fluids via a custom clamp‐on sensor. This device was designed to enable each of the four mechanisms for biomarker quantification. Further on, we calibrated the device to ensure reproducible and accurate measurements, which were validated by LC‐MS analysis. Inspired by the need for real‐time biomarker measurements prior to transplant surgery and during ex vivo perfusion, our device was designed to be compatible with applications in an operating room without compromising sterility and to measure biomarker concentrations with at least the accuracy of state of the art methods. A modular design facilitated the integration of additional light sources, enabling the measurement of additional biomarkers. The custom clamp‐on probe was designed so that light enters the flow cell perpendicularly to the spectrometer cable, minimizing direct stimulation of the photodetectors. All components were housed in a compact container, allowing for convenient transport and sterilization. To make our device more user friendly, we designed our own software tool that measures and reports results in real‐time on a graphical user interface. We also developed an iOS application that clinicians could use on their mobile phones, freeing surgeons to perform other duties during organ perfusion and assessment Figure S4 (Supporting Information).

**Figure 2:**
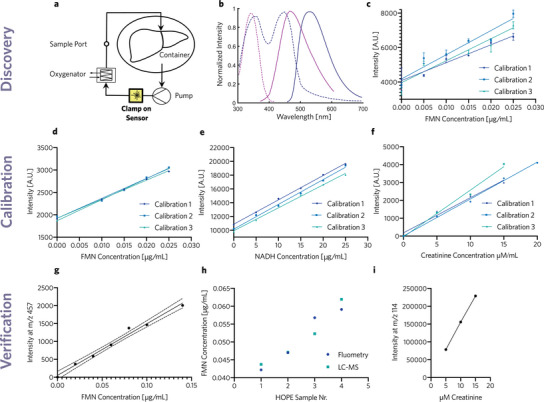
a) Perfusion setup for hypothermic oxygenated liver perfusions, featuring a peristaltic pump, an oxygenator, a container, and our spectrofluorometric sensor. b) Absorbance (dashed) and emission (solid) spectra for NADH (purple) and FMN (blue) allow for spectrofluorometric measurements.^[^ ^14^, ^15^ ^]^ c) Plate reader measurements of FMN solutions based on the method of Muller et al.,^[^ ^16^ ^]^ showing substantial sample‐to‐sample variability. d) Intensity, measured by the spectrometer, as a function of the perfusate FMN concentration, serving as a calibration for our device. FMN was excited at 405 nm and emission was measured at 530 nm. e) Spectrometer intensity as a function of perfusate NADH concentrations as calibration for our device. NADH was excited at 385 nm and emission was measured at 460 nm. f) Spectrometer intensity as a function of dialysate creatinine concentration as calibration of our device. g) Liquid chromatography mass spectrometry (LC‐MS) intensity peaks at m/z: 457 Da for different FMN concentrations to validate concentrations of calibration solutions. h) Fluorescent measurements were compared with LC‐MS intensity at 457 Da in collected HOPE perfusate samples, validating real‐time spectrofluorometric data with state‐of‐the‐art LC‐MS data in a biological fluid. i) LC‐MS intensity peaks at m/z 114 Da for different creatinine concentrations to confirm concentrations of calibration solutions.

**Figure 3:**
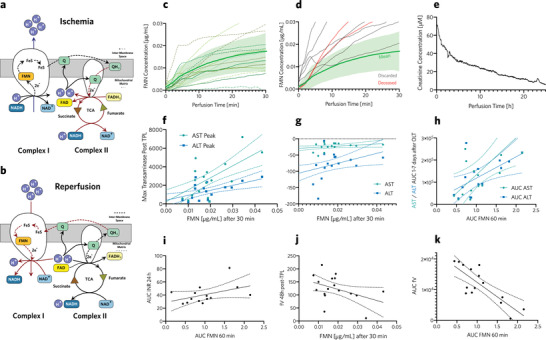
Mitochondrial damage: Respiratory chain during ischemia a) and reperfusion b). c) Real‐time measurements of change in FMN concentration in perfusate during HOPE of grafts that were transplanted, indicating progress of ischemic reperfusion injury in real‐time. Mean FMN concentration (green bold line) and 95% confidence region (green shaded area) of successfully transplanted grafts. d) Real‐time FMN concentration during HOPE of discarded grafts (gray lines), and grafts transplanted into patients who deceased after transplantation (red lines). Mean FMN concentration (green bold line) and 95% confidence region (green shaded area) of successfully transplanted grafts. e) Blood creatinine concentration, measured during normothermic liver perfusion. f) Peak inflammation marker concentrations after a transplant, compared to FMN concentrations (µg mL^‐1^), 30 min after HOPE began. Higher FMN concentrations during HOPE correlated with peak transaminase levels after transplantation, which indicated increased hepatic inflammation. g) Exponent of transaminase decay compared with FMN concentration at 30 min of HOPE. Lower FMN concentrations during HOPE correlated with faster decay of transaminase levels indicating more rapid patient recovery. h) Area under the curve (AUC) compared between AST, ALT (days 1–7 after transplant) and FMN within the first 60 min of HOPE. This correlation shows that patients with grafts with lower FMN concentrations were exposed to less hepatic inflammation after liver transplantation. i) Correlation of AUC of released FMN during the first 60 min of HOPE and INR during the first 24 h after liver transplant. As a reference, an average INR of 1 during the first day would be equivalent to an AUC INR 24 h of 24. Hence, grafts with higher FMN values exhibited increased INR after transplantation, thereby indicating slower recovery of hepatic function. j) AUC of factor V within days 1–7 after transplant compared to AUC of FMN within the first 60 min of HOPE. This showed that patients with low FMN grafts had improved coagulation factor synthesis after transplantation, indicating better organ function. k) Interpolated fV value, 48 h after transplantation, showing increased liver function for patients with low FMN grafts.

We demonstrated our approach with two examples, measurement of FMN during HOPE and measurement of creatinine clearance in whole blood by using a hemodialysis bypass and measuring creatinine in waste dialysate Figure 3. For HOPE measurements, a sterile flow cell (Medica, M90392) was integrated into the perfusion circuit and connected to two light sources via our custom clamp‐on probe. Our device was calibrated by measuring signals of Belzer MPS solutions with known concentrations of FMN. These measurements were robust over three independent calibrations **Figure** **2**d. NADH was also quantified in Belzer MPS with high accuracy Figure 2e. There was negligible cross‐talk between FMN and NADH in mock‐up perfusions Figure S6 (Supporting Information). Further, the fluorescent signal of bilirubin was negligible at concentrations above those found in standard HOPE perfusate. Even for much higher concentrations in the range of 100 µM no significant emission could be found at 530 nm (Figure S11, Supporting Information). To conclude the development of a real‐time biomarker assay, measurements have to be confirmed with an independent method as the last and fourth step. The spectrophotometric measurements of FMN in HOPE perfusate were confirmed by LC‐MS Figure 2h. Similarly, the device was calibrated with dialysate solutions of known creatinine concentration Figure 2f. These dialysate samples were also analyzed with LC‐MS to validate the spectrophotometric measurements Figure 2i.

### 2.2 Biomarkers in Liver Transplantation

Due to a persistent donor‐organ shortage transplant surgeons increasingly resort to liver grafts from donors after circulatory death (DCD).^[^ ^17^, ^18^ ^]^ However, these grafts are more susceptible to ischemia, which is one of the main mechanisms in the pathogenesis of liver injury and primary non‐function.^[^ ^19^, ^20^ ^]^ Increased ischemic damage results in a higher risk of non‐anastomotic hepato‐biliary strictures after liver transplantation.^[^ ^21^, ^22^ ^]^ Ischemic damage is exacerbated upon reperfusion after transplant, which is commonly referred to as ischemic reperfusion injury (IRI).^[^ ^23^, ^24^ ^]^ Several clinical strategies are being investigated to understand and attenuate IRI.^[^ ^25^, ^26^ ^]^ One of the most promising approaches to ameliorate IRI is ex situ hypothermic oxygenated liver perfusion (HOPE) prior to transplantation.^[^ ^13^, ^24^, ^27^, ^28^, ^29^ ^]^ In a recent clinical study, HOPE decreased the risk of non‐anastomotic complications in DCD liver grafts with a median asystolic warm ischemia time of 11 min.^[^ ^29^ ^]^ While HOPE considerably improved DCD transplant outcome, there is currently no method to quantitatively assess organ quality during ex situ perfusion and estimate the amount by which IRI related risk has been decreased by HOPE prior to transplantation.

### 2.3 Liver Graft Assessment

Standard clinical practice for graft assessment includes a review of the history and medical records of the donor followed by a visual inspection of the graft in situ.^[^ ^30^ ^]^ As this analysis is not based on objective values but rather on experience and subjective perception, prognostic models have been developed to estimate the risk of graft failure.^[^ ^31^ ^]^ These models are known as risk indices and consider a multitude of factors, including donor age, sex, height, weight, race, and cause of death.^[^ ^32^, ^33^ ^]^ Despite these indices, no robust metrics exist for graft assessment prior to transplantation. Therefore, additional measures, such as biomarkers, are needed to improve organ assessment prior to transplantation. In addition to the reduced risk of IRI, machine perfusion, including HOPE, provides an opportunity to measure biomarkers in the organ perfusate prior to transplantation. For example, normothermic machine perfusion (NMP) enables the assessment of metabolic function, which can be observed via lactate clearance, glucose evolution, and pH regulation.^[^ ^30^, ^34^, ^35^, ^36^ ^]^ Here, bile produced by a liver during normothermic perfusion with pH > 7.5 served as a putative biomarker that correlated with cholangiopathy.^[^ ^34^ ^]^

### 2.4 FMN as a Marker of Mitochondrial Injury

One potential biomarker of general graft quality is a measure of mitochondrial damage, which occurs upon collection of the donor graft. The respiratory chain enters an ischemic state, where the citric acid cycle reverses **Figure** **3**a.^[^ ^37^, ^38^ ^]^ This consumes two electrons, provided by complex I, thereby deactivating complexes III and IV and ceasing proton transport across the mitochondrial membrane.^[^ ^38^ ^]^ This results in a decreased proton gradient across the membrane, a deficit of ATP, and an associated compensation via ADP conversion to ATP.^[^ ^27^, ^38^ ^]^ During this process, mitochondria accumulate NADH, AMP, and succinate.^[^ ^27^, ^37^, ^38^, ^39^, ^40^, ^41^ ^]^ Upon reperfusion, ATP synthase catalyzes ADP production from AMP, which requires fewer protons and increases the proton gradient across the membrane to an extent that protons enter through complex I into the mitochondrial matrix.^[^ ^38^ ^]^ This reverse flow of protons is facilitated by reverse electron flow. As a consequence of reverse electron flow, flavin mononucleotide (FMN), a part of complex I, is released together with reactive oxygen species, permanently destroying complex I.^[^ ^27^, ^37^, ^39^, ^40^, ^41^, ^42^ ^]^ The released FMN enters the extracellular space and mixes with the perfusion liquid. Therefore, the presence of FMN in the perfusate during machine perfusion serves as a surrogate marker of mitochondrial damage. FMN and NADH are fluorescent in their oxidized and reduced forms, allowing for convenient fluorometric measurements Figure 2b.^[^ ^15^ ^]^

In the clinic, the presence of FMN in the perfusate has been related to IRI upon organ transplantation.^[^ ^16^ ^]^ Elevated FMN concentrations predicted severe allograft dysfunction and early graft loss.^[^ ^16^ ^]^ Further, FMN concentration correlated with post‐operative lactate clearance and the synthesis of coagulation factors – lower FMN values were indicative of better graft performance. These findings demonstrate the clinical potential of evaluating perfusate FMN concentration during HOPE; however, they relied on discrete fluorometric measurements of perfusate samples.^[^ ^27^, ^40^, ^43^ ^]^ Discrete measurements with lab‐grade plate readers lead to considerable sample‐to‐sample variability, compromising the accuracy and aggravating later data analysis Figure 2c. In addition, these measurements are time consuming, which can delay planned procedures, and require access to the perfusate, which introduces the risk of contamination. Further, it was not possible to observe FMN dynamics using discrete measurements and most of the data directly after reperfusion on the HOPE machine was not available. Continuous and robust data about graft quality are needed to better guide clinical decisions and further our understanding of ischemic reperfusion injury.

#### 2.4.1 Real‐Time Graft Assessment

To demonstrate the clinical utility of our spectrofluorometric method, FMN was continuously measured in 26 extended criteria donor (ECD) livers during HOPE Table S1 (Supporting Information). Real‐time measurements taken with the portable sensor correlated with the discrete samples and showed a strong correlation between values at 30 and 60 min. Therefore, the extent of ischemic damage and likelihood of related IRI could be estimated already after 30 min using the real‐time data.

Further, real‐time measurements provided more information about biomarker dynamics and, in the case of FMN, decreasing slopes or even a plateau indicated good organ quality while increasing slopes represented progressive ischemic damage. In the absence of real‐time measurements, the current recommendation is to take additional discrete measurements of FMN after 45 and 60 min to observe if the ischemic damage is progressing for all samples where the 30 min measurement indicated mitochondrial damage.^[^ ^40^ ^]^ With the portable sensor, the surgical team could assess organ quality based on real‐time measurements directly within the OR, accelerating the assessment period by up to one hour, discriminating transplantable grafts from non‐transplantable grafts **Figure** **3**c,d.

We identified seven livers with elevated FMN concentrations that were discarded because the corresponding recipient was deemed unfit for the anticipated level of IRI, based on the FMN measurements Figure 3d. Because only discrete measurements of FMN have been validated and reported in literature, discard decisions were based on discrete plate reader measurements that were collected in parallel Figure S13 (Supporting Information). The remaining 19 grafts were transplanted Figure 3c and patient blood samples were collected routinely after transplantation. Post‐transplant transaminase levels correlated with higher FMN concentrations after 30 min of HOPE Figure 3f. These results demonstrated that selecting grafts based on FMN mitigated the risk of hepatic inflammation and additional complications.^[^ ^44^ ^]^ To further study the dynamics of transaminase decrease, we compared data from exponential transaminase fits to FMN concentrations after 30 min of HOPE Figure 3g. Patients with higher FMN grafts took longer to normalize transaminase levels. To further use our continuous datasets, we calculated the area under the curve (AUC) for FMN over the first 60 min of HOPE, and compared it to the AUC of transaminase levels during the first seven days following transplantation Figure 3h. Increased AUC for FMN correlated with increased AUC of ALT and AST, indicating that overall mitrochondrial damage was a further indicator of organ function in vivo. In other words, patients with lower FMN grafts were exposed to less inflammatory responses after transplantation.

Since the liver produces coagulation factors, the international normalized ratio (INR) is commonly used to assess liver function, where a value larger than one indicates longer prothrombin times and therefore impaired liver function. To compare the recovery of INR values during the first 24 h after transplantation, we compared the AUC of INR over the first day with the AUC of released FMN during the first 60 min of HOPE Figure 3i. Patients that received grafts with higher FMN values showed higher prothrombin times than those with lower FMN values during perfusion. To assess the role of the liver in blood coagulation more specifically, factor V synthesis is often used as a marker for liver function; factor V values 48 h after orthotopic liver transplantation are correlated with HOPE FMN concentrations Figure 3j. Patients who received livers with lower FMN concentrations showed higher factor V synthesis suggesting increased organ function. The AUC was calculated for coagulation factor V synthesis Figure 3k, which also correlated with the AUC for FMN.

### 2.5 Creatinine as a Biomarker for Renal Failure

To further expand the utility of real‐time biomarker measurements for the detection of non‐fluorescent markers, we measured creatinine levels in perfused livers as an indicator of insufficient renal clearance or impaired hemodialysis. Acute renal failure and chronic kidney disease are among the most prevalent complications following liver transplantation.^[^ ^45^, ^46^ ^]^ They are caused by preoperative conditions, such as hepatorenal syndrome, kidney impairment that is usually associated with cirrhosis, perioperative complications, and post operative patient recovery.^[^ ^46^, ^47^ ^]^ Renal function is commonly assessed by estimating the glomerular filtration rate. It can be calculated by measuring clearance of injected inulin or plasma creatinine.^[^ ^48^, ^49^, ^50^ ^]^ Higher serum creatinine concentrations were also found as a consequence of mild renal impairment due to COVID‐19 infection with mild pneumonia.^[^ ^51^ ^]^ In the field of abdominal organ transplantation, creatinine was monitored in patients following liver transplantation and peak serum creatinine was associated with increased mortality.^[^ ^52^, ^53^ ^]^ Even with hemodialysis as a renal replacement therapy, mortality increased for patients who were shown to have insufficient renal function after a liver transplantation.^[^ ^54^ ^]^ Therefore, it is even more important to diagnose impaired kidney function as early as possible and gain further understanding of the pathogenesis of post‐transplant associated renal failure.

#### 2.5.1 Real‐Time Patient Monitoring

The previously described four key steps in the development of real‐time biomarker assays Figure 1 were also applied to measure creatinine in waste dialysate. Hemodialysis is commonly used to clear the blood of patients with impaired renal function, but is also applied during ex vivo perfusions with blood‐based perfusates.^[^ ^35^ ^]^ Waste dialysate contains molecules from patient blood, including creatinine, and therefore reflects their concentrations in blood, which can provide non‐invasive and quantitative information on biomarker values Figure 1. The gold standards for measuring creatinine concentrations are gas chromatography and mass spectrometry.^[^ ^55^ ^]^ Creatinine concentrations can then be used to calculate creatinine clearance and the glomerular filtration rate, which indicate renal function. However, these methods again preclude real‐time data collection and require access to the biological fluid, introducing the risk of contamination. As spectrofluorometric measurements were not directly possible in whole blood, we performed real‐time creatinine measurements on waste dialysate, which equilibrates with the concentration in the blood. We measured creatinine values in an ex vivo perfused hemi‐liver on a hemodialysis loop Section S5 (Supporting Information). In order to measure creatinine concentrations with high accuracy, we adapted the protocol by Lewinska et al.^[^ ^56^ ^]^ for continuous measurements and the spectrophotometric measurements were verified by LC‐MS Figure 2i. We monitored the creatinine concentration in the blood‐based perfusate of the aforementioned human hemi‐liver over the first 24 h of normothermic perfusion Figure 3e. Creatinine was effectively cleared by our hemodialysis filter suggesting that a dialysis bypass flow rate of 0.2 L min^−1^ blood and a dialysate flow rate of 200 mL h^−1^ allow for efficient blood filtration. A similar approach could be applied to measure creatinine concentrations in patients undergoing renal replacement therapy in a clinical setting or to assess biomarker concentrations in other dialysis settings.

## 3 Conclusion

We developed a spectrofluorometric method to measure biomarkers non‐invasively within biological fluids and in real time. This method provided high accuracy for both, fluorescent and non‐fluorescent biomarkers and can be used in a broad range of applications, such as organ assessment in ex vivo perfused organs, in vivo patient monitoring, and cell culture media measurements for in vitro experiments. We used our method in the clinic for continuous measurements of FMN, a marker of mitochondrial damage and reperfusion injury, in 26 ECD human liver grafts during HOPE. The online FMN measurements of reperfusion injury improved organ assessment directly in the operating room while providing additional valuable data to transplant surgeons. Real‐time FMN concentrations at early time points (30 min) during HOPE correlated with later time points (60 min) allowing surgeons to assess graft quality earlier and more reliably. Further, rapid changes in FMN after 30 min indicated progressive reperfusion injury and mitochondrial damage, and served as a sign for poor organ quality. Based on discrete plate reader measurements of FMN, seven grafts were discarded as the transplant team anticipated that they would put patients at an untenable risk of complications. However, our real‐time measurements suggested that at least one additional graft could have been transplanted, according to our criteria. An additional 19 grafts, which would have been otherwise discarded as they came from extended criteria donors, were transplanted based on the FMN measurements, that indicated suitable graft quality following HOPE. In the transplanted patients, the FMN data (absolute concentration at 30 min and total AUC) correlated with transplant outcome and post‐transplant transaminase values, demonstrating the utility of this approach to predict patient outcome. Two patients deceased following liver transplant that would have been difficult to predict based on discrete measurements Figure S13 (Supporting Information). However, our real‐time measurements demonstrated clear deviations from our confidence region for these grafts, and these organs would not have been transplanted taking into account the improved data from our real‐time measurements. The general approach to monitor donor graft quality using a non‐invasive spectrophotometric device extends beyond liver transplantation. Mitochondrial function is critical to every transplanted organ and real‐time measurements of FMN would be advantageous during heart, lung, and kidney perfusion. By leveraging a dialysis bypass to measure biomarkers in waste dialysate, FMN and other biomarkers can also be measured during normothermic perfusions with blood‐based perfusate Figure S21 (Supporting Information). This approach offers transplant surgeons new opportunities to assess grafts not only on the organ scale but also at the sub‐cellular level. Further, our FMN assessment reflects the condition of the whole organ, while histological reports, which are commonly used for assessment, only represent a small fraction of the tissue. Benchmarks for reperfusion injury in transplanted organs offer increased patient safety and are a first step toward more objective graft assessment. Since mitochondrial damage and its amelioration is the subject of many ongoing studies, measurements could also be performed in cell and organoid culture, where cell medium is circulated through a flow cell.

Beyond FMN and markers of mitochondrial damage, the general approach to monitor biomarkers in biological fluids presents many opportunities to improve clinical care. In order to show the feasibility of real‐time biomarker measurements of non‐fluorescent biomarkers, we measured creatinine concentrations in waste dialysate, assessing hemodialysis during normothermic ex vivo perfusion. This also demonstrated that indirect measurements could be performed with a non‐transparent perfusate, whole blood, and the same technology could be used to monitor transplant or dialysis patients during the course of their treatment. By monitoring patients who undergo hemodialysis, our technology forms the basis for further marker discovery, patient assessment, and improved clinical care. Future applications of our real‐time biomarker assay, could also include FRET‐based measurements of biomarkers or in situ enzyme activity by adding complementary molecules to the biological fluid.

Overall, we demonstrated the development of spectrofluorometric devices that allow for continuous real‐time measurements of biomarkers in clinics, where discrete sampling and elaborate laboratory analysis are still predominant. Moving toward continuous monitoring provides clinicians with more data that can be saved on patient data management systems where it is instantly available to supervising clinicians who can follow other duties at the same time. As our approach can be used to measure a variety of fluorescent and non‐fluorescent biomarkers, we hope to accelerate broader use of biomarkers, that were discovered but are not routinely used in clinics due to elaborate measurements and insufficient time resolution. In particular, we demonstrated the clinical utility of simple spectrophotometric measurements of key biomarkers such as FMN, NADH, and creatinine to address the clinical need to improve donor graft assessment and patient care. We established new standards for accuracy of biomarker measurements in biological fluids, providing high‐precision measurements to clinicians and researchers without expensive laboratory equipment, harmonizing measurement units and improving comparability. Further, portable and online devices save costs and allow clinical staff to focus on other tasks during biomarker assessment. This is especially enabled by our mobile application since clinicians can now access real‐time biomarker data on their smartphone, which saves time and facilitates transplant coordination. We hope that our approach for the development and implementation of real‐time biomarker assays will inspire other researchers and clinicians to apply this method to their biomarkers of interest, expanding its potential to transform patient care. In total, we introduced an essential tool for clinical use in the field of organ transplantation that also opens new avenues in improved patient diagnosis and therapy, biomedical research, and biomarker discovery.

## 4 Experimental Section

### Sensor Specifications

The portable sensor was built by connecting an Avantes HP‐LED405 to an Avantes StarLine spectrometer. The latter was connected to a Laptop via USB where a custom Python code was running. The temporal resolution was limited by the chosen integration time in the spectrometer settings, the number of connected light sources, and the protocol speed by which the light sources and spectrometer were operated. A sensor integration time of 2 s was chosen since it maximized signal intensity while still allowing measurements over a broad dynamic range without saturating the sensor. In the sensor design with two light sources, this study could measure FMN concentration every 11 s. Slight variations in the initial measurements were observed in pure Belzer MPS samples; however, the slope of the signal always remained the same for increasing sample concentrations. Hence, a simple calibration was required before connecting the liver, where an offset <0.02 µg mL^−1^ was calculated by averaging ten samples with pure Belzer MPS. Measurements were not affected by sample temperature over the range of 2 to 25 °C. Similarly, the flow rate did not affect measurement accuracy. With a lower detection limit (LOD) of 4.4 nM for FMN, this study's detection limit was in the range of the LOD of quantitative LC‐MS^[^ ^57^ ^]^ and approximately ten times lower than previous discrete fluorometric measurements in biological samples.^[^ ^58^ ^]^

### Custom Software

A custom software was developed for consistent and precise measurements. The software controlled the light sources, the spectrometer, filtered and converted measured data, and subsequently saved concentrations with a time stamp into a PostgreSQL database. To account for changes in ambient light or changes of sensor sensitivity due to thermal effects, a dark measurement was acquired for every measurement by automatically turning off the LED. The darkfield spectrum was then subtracted from the next measurement yielding robust measurements even if the light conditions varied. To provide real‐time data, measurements were triggered automatically and continuously by the software. A simple graphical user interface provided real‐time data, that allowed clinicians to follow changes of marker concentrations directly in the OR. Further, an iOS application with Swift that showed real‐time data on a mobile phone was developed. Measurements were uploaded to a cloud‐based PostgreSQL database, from where it could be accessed with the mobile App.

### Sensor Calibration

Calibration curves for FMN measurements were produced by circulating 50 mL of fresh Belzer MPS with defined quantities of FMN. Once the signal reached a steady state and the fluid was properly mixed, the signal was averaged over 5 min Figure S5 (Supporting Information). Samples from the same calibrations were also collected and were analyzed with a Cytation seven plate reader, following current protocols used in the clinic.^[^ ^59^ ^]^ Three samples were measured per concentration and per calibration, and the plate reader measurements of the same sample had more variability than our real‐time measurements Figure S9 (Supporting Information).

### HOPE

Donor livers were flushed via the portal vein in situ with cold Institute George Lopez (IGL) solution to minimize warm ischemia time and were subsequently transported on ice to the Swiss HPB center at the University Hospital Zurich. Grafts were cannulated and the portal vein was connected to a VitaSmart Bridge to Life HOPE system. Hypothermic perfusion was carried out for at least 1 h with a maximum flow rate of 250 mL min^−1^ and a maximum pressure of 3 mmHg. Three liters of Belzer MPS (developed by Belzer and Southard at the University of Wisconsin) were circulated with an oxygen partial pressure larger than 100 kPa. The temperature was kept around 5 °C by cooling the liver container with ice.

Patients received an orthotopic liver transplant after HOPE and more details about transplanted organs are given in Table 1 (Supporting Information). Details about deceased patients and data processing was provided in the Sections S4.3–4.6 (Supporting Information). Experiments related to HOPE were approved by local authorities (Cantonal Ethics Committee Zurich KEK no. 2019‐01000) and were carried out with the full, informed consent of the subjects.

### Discrete FMN Measurements

Discrete perfusate samples were collected after 5, 10, 15, 30, and 60 min of HOPE, following current practice in the clinic. These samples were analyzed outside of the operating room with a Cytation seven plate reader. A high FMN concentration (3000–4000 A.U.) was regarded an indicator of high ischemic damage and an initial criterion to discard the organ. While the patient was waiting for anesthesiologists to start induction, a surgical resident performed aforementioned laboratory analysis and reported results to the surgical team 1.5 h after the start of perfusion.

### Online FMN Measurements

A custom clamp‐on sensor was connected to the HOPE tube set before priming. After priming, the sensor was calibrated with pure Belzer MPS in the tubes to account for signal offsets due to variance in individual flow cells. FMN was excited at 405 nm and fluorescent emission was measured at 530 nm. Once the liver was connected to the perfusion circuit, FMN concentrations were displayed live on a separate screen. Data was recorded in a database and measurements were continued until the graft was removed from HOPE upon implantation or disposal.

### Online Creatinine Measurements

Waste dialysate was collected in a bypass at a rate of 20 mL h^−1^ with a peristaltic pump and mixed with a 2 mol L^−1^ NaOH solution (5 mL h^−1^) and a reagent solution (5 mL h^−1^). The reagent was previously mixed in a 50 mL falcon tube by adding 45 mL of 1,4‐butanediol, 5 mL of milli‐Q water, 204 µL of hydrogen peroxide and 265 mg of 3,5‐dinitrobenzoic acid. All solutions were propagated by peristaltic pumps in Heidelberger extension lines, which were connected to a single three‐way valve. After this, all solutions flowed in the same tube and the solution was mixed with a magnetic stirrer. Connection lines were chosen in a way that the solution took 30 min to reach the cuvette to ensure complete chemical reaction. The fluorescent compound, produced in the aforementioned reaction, was excited with a wavelength of 405 nm and emission was measured at 475 nm, respectively.

## Conflict of Interest

P.A.C., P.D., and M.H. are co‐inventors on patents related to biomarker analysis during organ perfusion. P.A.C. founded a company, QRSens, to commercialize the technology.

## Author Contributions

F.H. designed the experiments. F.H. and J.B. built the device. F.H., M.H., and J.B. took measurements during HOPE. J.E. and R.X.S.D.S. performed HOPE and collected clinical data. F.H. analyzed data. F.H., M.W.T., and L.C. wrote the manuscript. P.A.C., P.D., M.W.T. and M.H. supervised the project.

## Supporting information

Supporting Information

## Data Availability

The data that support the findings of this study are available on request from the corresponding author. The data are not publicly available due to privacy or ethical restrictions.
