# Supplementary material for: A Spectrofluorometric Method for Real‐Time Graft Assessment and Patient Monitoring
Source: Adv Sci (Weinh). 2023 Jun 2;10(23):2301537. doi: 10.1002/advs.202301537 (PMC10427358; doi:10.1002/advs.202301537)
Supplement: Supplementary file 1 — Supporting Information [file ADVS-10-2301537-s001.pdf]

ADVANCED SCIENCE
Open Access

Supporting Information

for *Adv. Sci.*, DOI 10.1002/advs.202301537

A Spectrofluorometric Method for Real-Time Graft Assessment and Patient Monitoring

Florian Huwyler, Janina Eden, Jonas Binz, Leslie Cunningham, Richard X. Sousa Da Silva, Pierre-Alain Clavien, Philipp Dutkowski, Mark W. Tibbitt* and Max Hefti**

ADVANCED SCIENCE Open Access
www.advancedscience.com

Electronic Supporting Information

A Spectrofluorometric Method for Real-Time Graft Assessment and Patient Monitoring

Florian Huwyler [1,2,3], *Janina Eden* [2], *Jonas Binz* [1], *Leslie Cunningham*[1,2,3], *Richard X. Sousa Da Silva* [2,3], *Pierre-Alain Clavien* [2,3], *Philipp Dutkowski* [2] ✉, *Mark W. Tibbitt* [1,3] ✉, *Max Hefti* [3] ✉

✉ e-mail: **philipp.dutkowski@usz.ch**, **mtibbitt@ethz.ch**, **max.hefti@proton.me**
DOI: **10.1002/advs.202301537**

Contents

1 Proposed Mechanism for Mitochondrial Injury 2

2 Graphical User Interface 4

3 Calibration & Accuracy 4
3.1 Calibration in Dialysate 5
3.2 Cross-Talk with Bilirubin 6

4 Clinical Data 6
4.1 Clinical FMN Measurements 6
4.2 Transplanted Organs 7
4.3 Patient Death after Liver Transplantation 8
4.4 Evaluation of Post-Transplant Transaminase Levels 8
4.5 Evaluation of fV Synthesis after Transplantation 8
4.6 Post-Transplant Data over Time 9

5 Normothermic Perfusion of Partial Liver for Real-Time Creatinine Measurement 9

6 Normothermic Perfusion of Discarded Grafts 11

7 FMN Measurement During Normothermic Perfusion 12

Affiliations:
[1] Macromolecular Engineering Lab, Department of Mechanical and Process Engineering, ETH Zurich, Zurich, Switzerland
[2] Department of Surgery and Transplantation, Swiss Hepato-Pancreato-Biliary (HPB) and Transplant Center, University Hospital Zurich, Zurich, Switzerland
[3] Wyss Zurich Translational Center, ETH Zurich and University of Zurich, Zurich, Switzerland

1 Proposed Mechanism for Mitochondrial Injury

Based on literature review, we compiled and proposed a mechanism of action for the release of FMN as a consequence of reperfusion after an ischemic phase.[1–8]

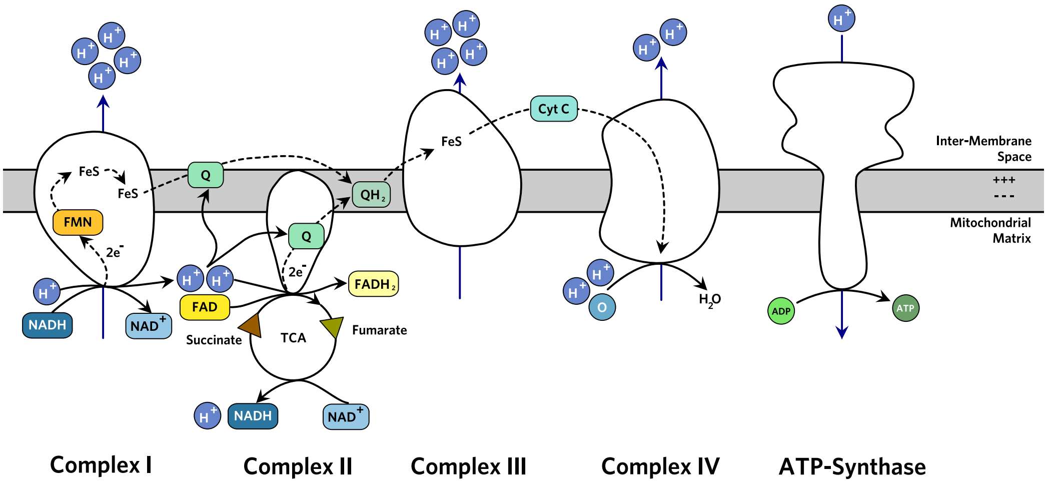

Supplementary Figure S1: Respiratory chain during normoxia

During normal physiological conditions, mitochondrial complexes I to IV create a proton gradient across the inner mitochondrial membrane. This gradient creates a proton flux through ATP-Synthases, which catalyze the production of energy rich ATP. At complex I, energy rich NADH is decomposed into $2H^+$ ions and NAD^+. This reaction releases two electrons which are transported along complex I. Electrons are transferred to ubiquinone (Q) via FMN and FeS and form together with 2 H^+ ions ubiquinol. This electron transfer initiates the movement of H^+ ions through complex I. Similarly, complex II reduces FAD as part of the citric acid cycle, thereby releasing 2 electrons. Ubiquinol transports electrons to complexes III and IV, which pump further H^+ ions across the membrane. Complex IV additionally requires oxygen to form water by combining two H^+ ions and oxygen. Due to the proton gradient, ATP-Synthase pumps protons back into the mitochondrion and synthesizes ATP, required for most vital cellular processes.

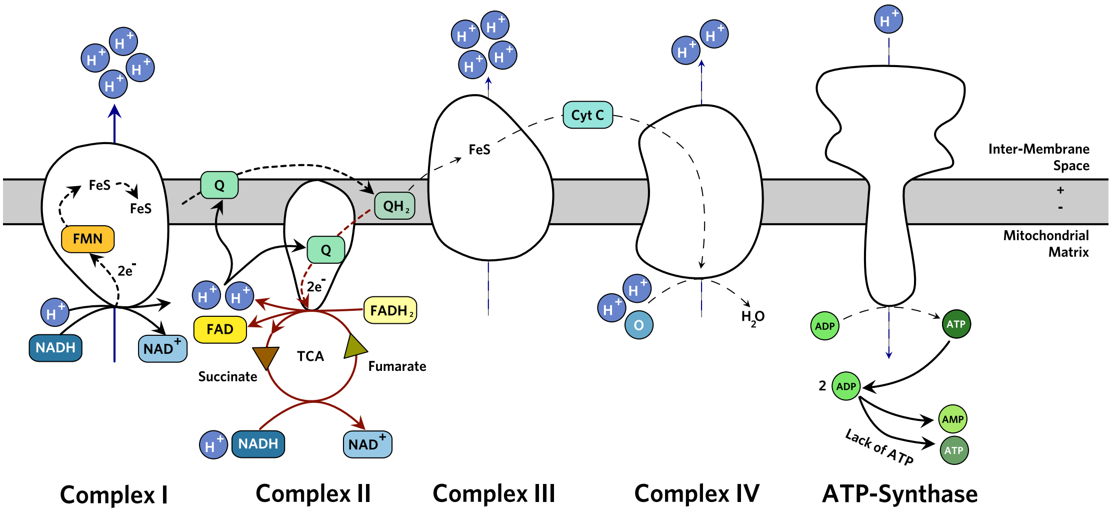

Supplementary Figure S2: Respiratory chain during ischemia

If cells are exposed to ischemia due to a lack of oxygen, biochemical processes adapt. Since complex I is not dependent on oxygen, it can operate as usual. Complex II on the other hand, runs reverse. As glycolysis requires NAD^+ to create 2 ATP per glucose, the citric acid cycle runs reverse thereby releasing 3 NAD^+ and oxidizing one $FADH_2$. This reduction requires 2 electrons which are provided by complex I. In other words, complex II features a reverse electron transport and deactivates complexes III and IV. Because complexes III and IV transport less H^+ ions, the gradient decreases and the ATP-Synthase produces less ATP. Therefore, ATPs are consumed more quickly and 2 ADPs are broken into an AMP and an ATP to make up for the lack of synthesized ATPs. This increases the concentration of AMPs. At the same time, succinate and NAD^+ concentrations increase and accumulate while NADH concentrations decrease within mitochondria.

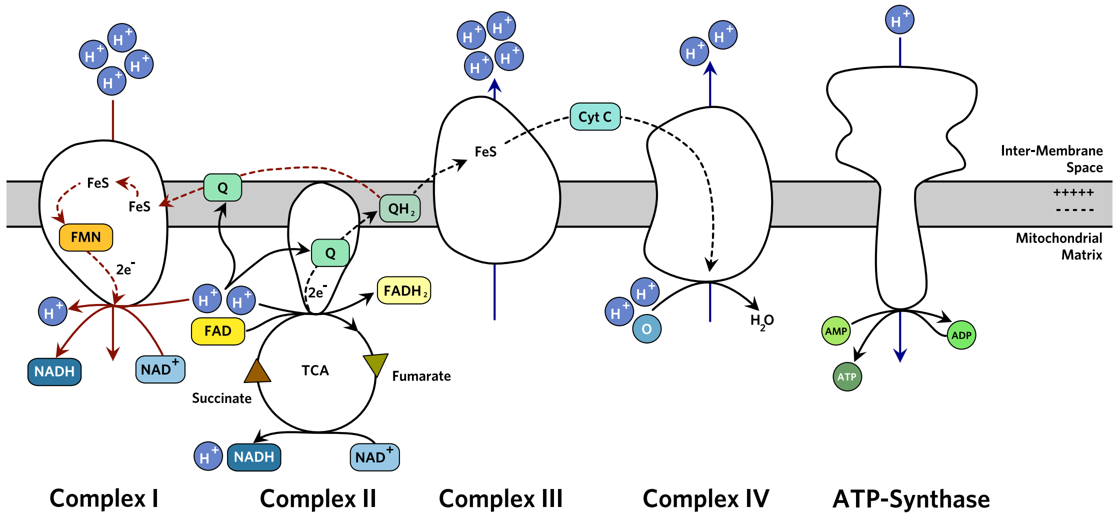

Supplementary Figure S3: Respiratory chain during reperfusion

Once, an organ is connected to a perfusion system again, oxygen is provided and transported into cells. Because of the accumulation of succinate, AMP and NAD^+, cells cannot go directly into normal operation. Since the concentration of NAD^+ is much larger than the concentration of NADH, NAD^+ is reduced. This requires two electrons which are provided by complex II. As these electrons pass complex I in the other direction, H^+ ions are sucked from the inter membrane space into mitochondria. Reverse electron transport due to the accumulation of succinate changes the redox potential of complex I thereby releasing FMN, which can pass through the membrane. Without FMN, complex I is deactivated. ATP concentration only recovers with a time lag as accumulated AMP have to be converted into ADP first.

2 Graphical User Interface

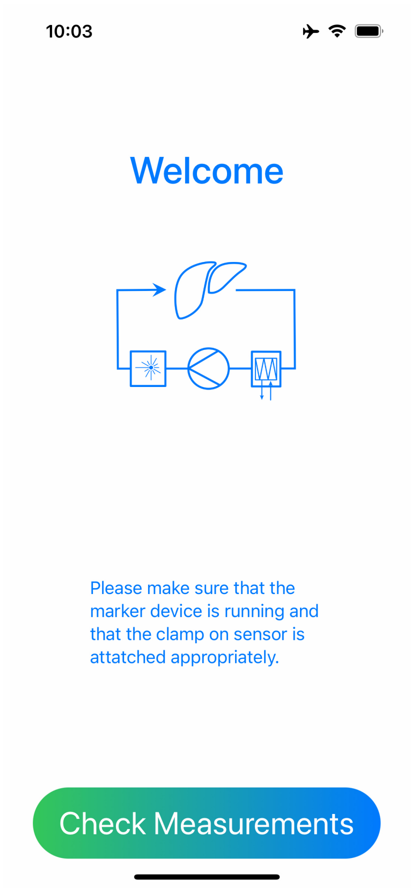

(a) Welcome Screen

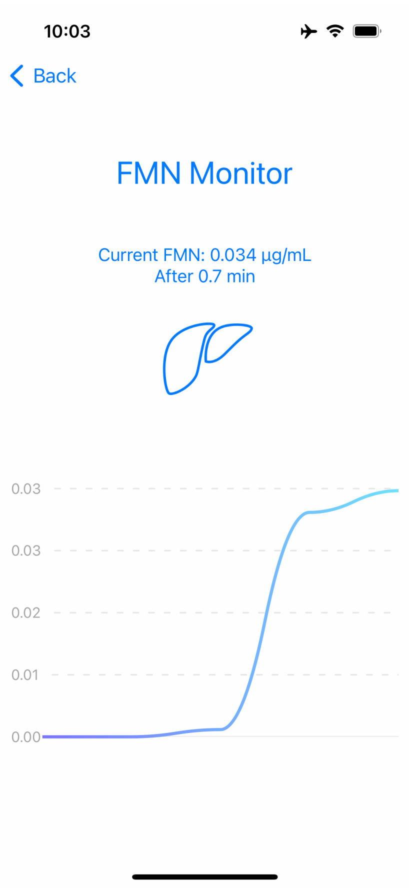

(b) Measurement Monitor

Supplementary Figure S4: iOS Application

3 Calibration & Accuracy

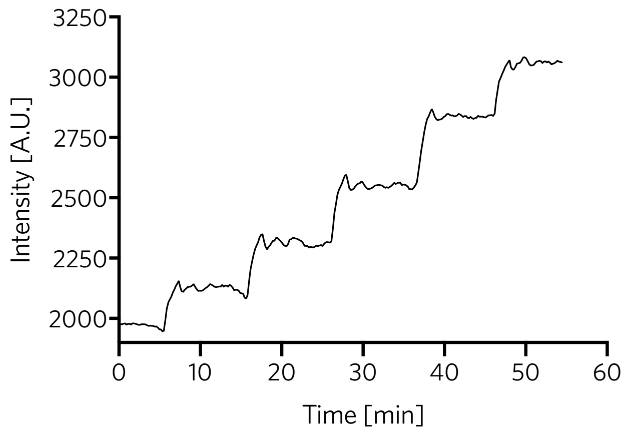

Supplementary Figure S5: Calibration curve of FMN in Belzer MPS in steps of 0.01μg/mL

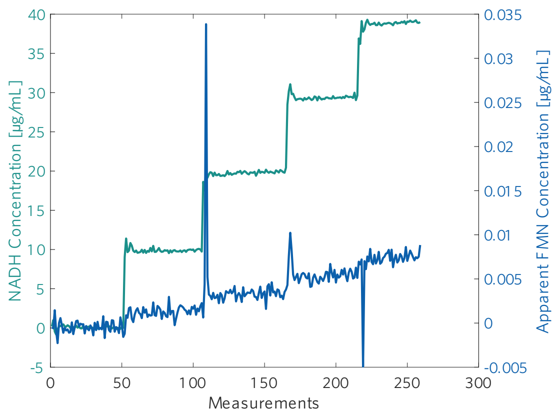

Supplementary Figure S6: Crosstalk between FMN and NADH in Belzer MPS

Based calibration curves, the following conversion formula could be found to calculate the FMN concentration based on intensity values from the spectrometer:

$$C_{FMN} = \frac{1}{47726.9}(I_{530}(t) - 2591.6) - C_{FMN}(0)$$

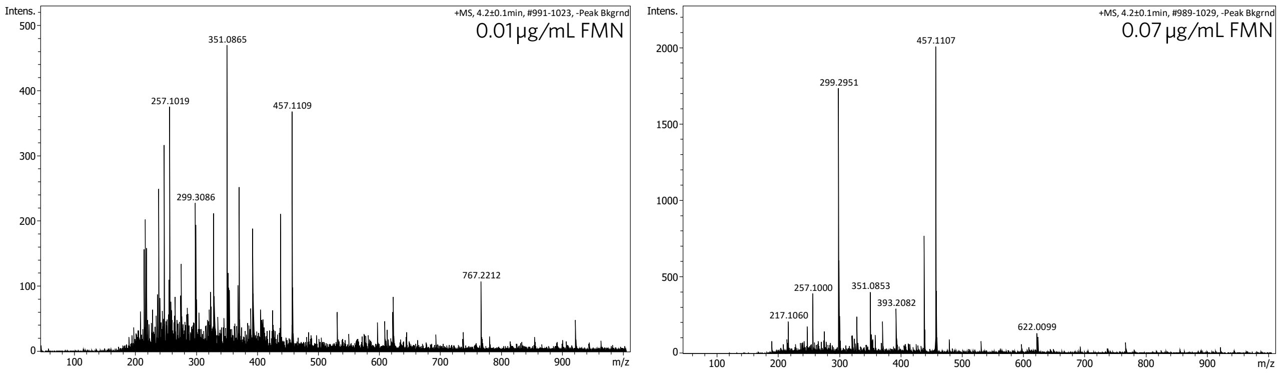

Supplementary Figure S7: LC-MS spectra for FMN samples in Belzer MPS for different concentrations.

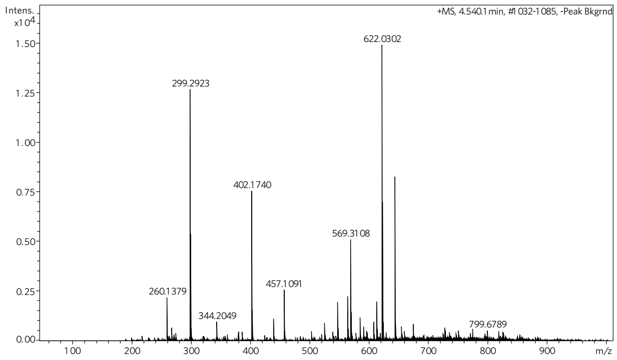

Supplementary Figure S8: LC-MS spectrum of collected HOPE perfusate sample

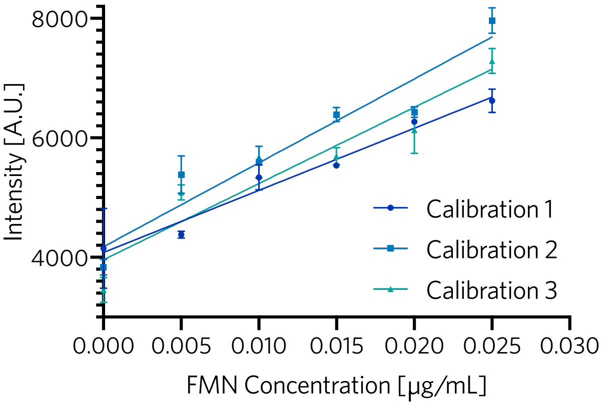

Supplementary Figure S9: Plate reader measurements that are currently in clinical use to determine FMN concentrations

3.1 Calibration in Dialysate

50 mL of dialysate were circulated while adding FMN and NADH to the circulated solution. Signals were averaged over at least 5 min and were correlated with the concentration of FMN and NADH respectively. Calibration curves were fitted analogously to calibrations done in Belzer MPS.

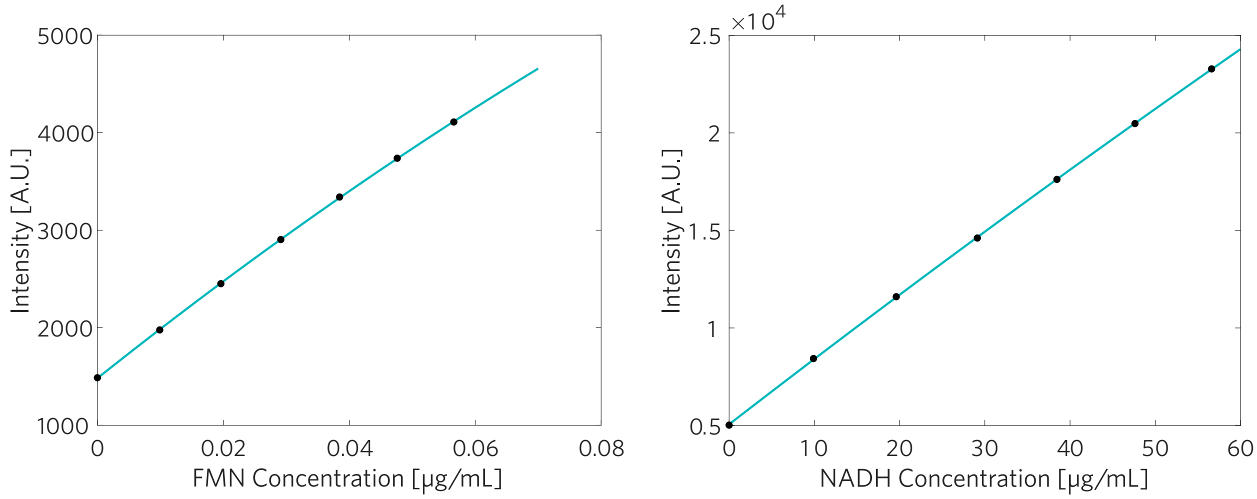

Supplementary Figure S10: Calibration of FMN and NADH in dialysate

3.2 Cross-Talk with Bilirubin

Bilirubin (14370-250MG, Sigma-Aldrich) was dissolved in dimethylsulfoxide (DMSO) to create a concentrated stock solution. The stock solution was diluted in 50 mL of aqueous solution and was circulated while measuring fluorescent spectra in a flow cell. In these experiments, the bilirubin concentrations were substantially higher than during standard HOPE, as demonstrated by the visible yellow color of a 35 μM solution shown in Supplementary Figure S12. Despite these high concentrations, no increase in fluorescence was visible at 530 nm while exciting the sample at 405 nm (Supplementary Figure S11).

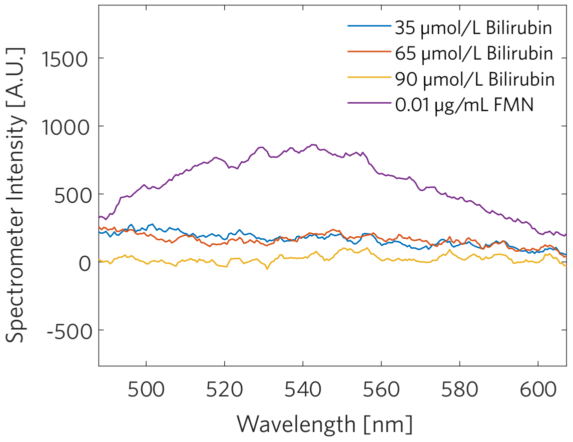

Supplementary Figure S11: Spectra of aqueous bilirubin and FMN solutions

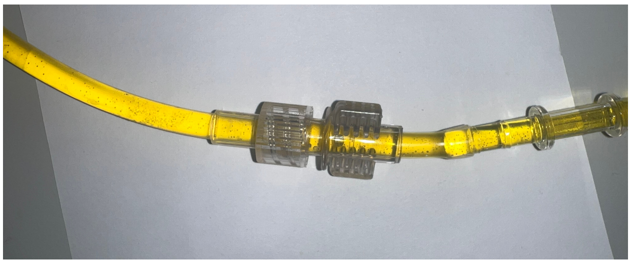

Supplementary Figure S12: Calibration with 35 μM bilirubin solution

4 Clinical Data

4.1 Clinical FMN Measurements

The following figure shows plate reader measurements of discrete samples that were used to accept or discard extended criteria donor grafts.

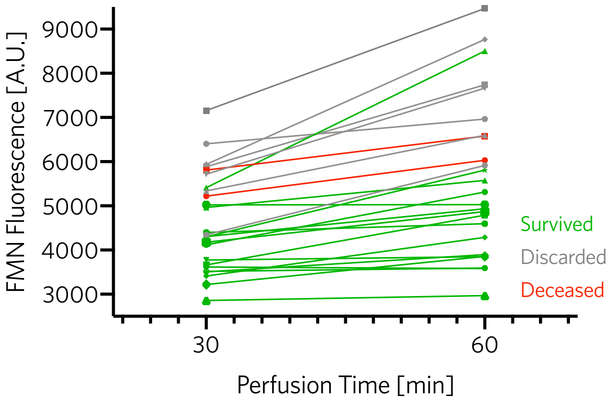

Supplementary Figure S13: Discrete FMN measurements after 30 and 60 min of HOPE

4.2 Transplanted Organs

Organ Nr.	TPL	Type	Donor Age	fWIT	Weight [g]	Steatosis
1	✓	DCD	29	28	2700	no biopsy
2	✓	DBD	28	N.A.	2340	no biopsy
3	✓	DCD	26	21	2200	<5% (micro/macro)
4	×	DCD	-	-	-	no biopsy
5	✓	DCD	70	18	1727	no biopsy
6	✓	DCD	23	23	2052	<5% (micro/macro)
7	✓	DBD	11	N.A.	854	no biopsy
8	✓	DBD	50	N.A.	1900	no biopsy
9	×	DCD	-	-	3200	no biopsy
10	×	DCD	-	-	2390	no biopsy
11	×	DCD	-	-	-	no biopsy
12	✓	DCD	75	16	1215	no biopsy
13	✓	DCD	47	33	2366	no biopsy
14	✓	DCD	48	22	1845	no biopsy
15	×	DCD	-	-	1712	no macro, 5% micro
16	✓	DCD	71	39	1350	no biopsy
17	×	DCD	-	-	1215	no biopsy
18	✓	DBD	76	N.A.	1824	20% macro
19	✓	DBD	80	N.A.	1800	no biopsy
20	✓	DCD	64	22	1804	no biopsy
21	✓	DCD	55	28	1635	5% macro
22	✓	DCD	21	19	1350	no biopsy
23	×	DCD	-	-	2399	no biopsy
24	✓	DCD	83	25	1995	25% (macro/micro)
25	✓	DCD	53	22	1304	no biopsy
26	✓	DCD	73	15	1284	15% macro

Table 1: Liver grafts - Additional information

4.3 Patient Death after Liver Transplantation

- Nr. 2: Patient died on the day of transplantation due to a non patent portal vein.
- Nr. 13: Patient recovered after TPL with AST < 50 and INR = 0.9 after 6 days. However, the patient underwent exitus letalis 4 month after TPL due to pneumonia.
- Nr. 21: Patient died 8 days after transplantation with AST = 435, ALT = 360, INR = 1 and fV = 103 due to a necrotic pancreatitis. The patient was treated with hemodialysis starting one day after transplantation.
- Nr. 24: Patient suffered from primary non-function two days after transplantation and was retransplanted. One day after retransplantation, the patient died due to another primary non-function.

4.4 Evaluation of Post-Transplant Transaminase Levels

All transaminase datapoints were fitted with a simple exponential model, as shown in equation 1, where t denotes the time after transplantation in hours, A denotes the amplitude of the exponential decay and B denotes the rate at which transaminase levels decreased.

$$AST(t) = A_1 \cdot \exp(B_1 \cdot t) \qquad ALT(t) = A_2 \cdot \exp(B_2 \cdot t) \tag{1}$$

To calculate the time it takes until AST values drop below 3000 U/L, we used the same exponential fit and equation 2.

$$t_{3000} = \frac{\ln(3000/A_1)}{B_1} \tag{2}$$

4.5 Evaluation of fV Synthesis after Transplantation

Measured fV values were interpolated with a second order polynomial fit to compare values at the same time point.

$$fV(t) = A_1 \cdot t^2 + A_2 \cdot t + A_3 \tag{3}$$

4.6 Post-Transplant Data over Time

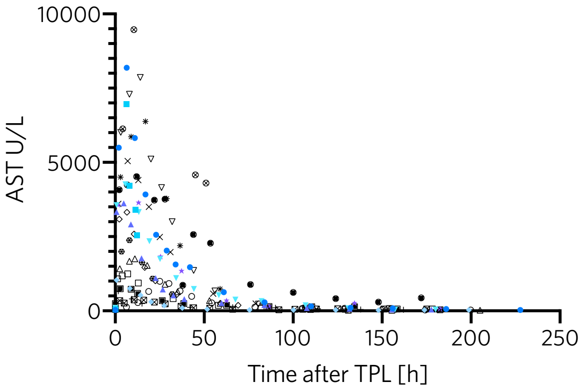

Supplementary Figure S14: AST data of all transplanted patients

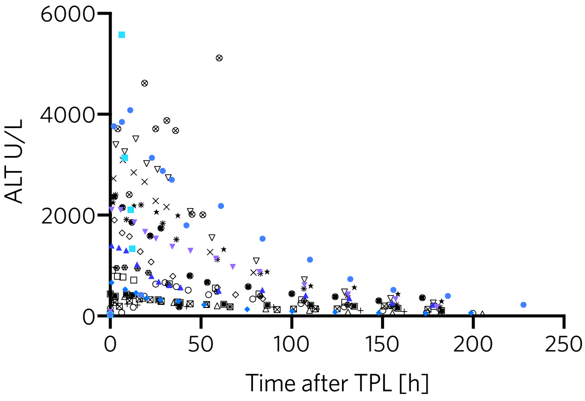

Supplementary Figure S15: ALT data of all transplanted patients

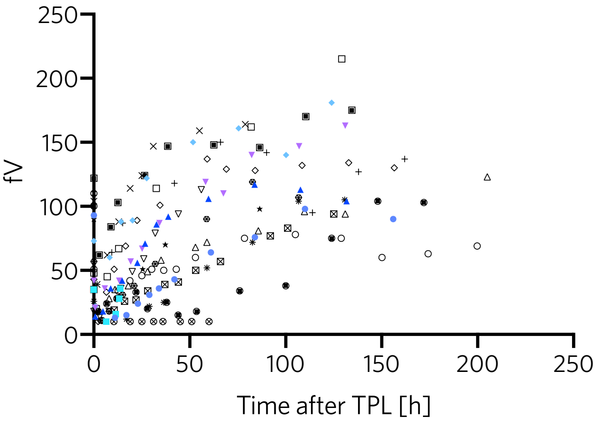

Supplementary Figure S16: fV data of all transplanted patients

5 Normothermic Perfusion of Partial Liver for Real-Time Creatinine Measurement

A 606 g right hemi-liver was perfused at 37 °C with 3L of whole blood with a haematocrit of 30% for 7 days (**Supplementary Figure S19**) . The liver was connected to a partially oxygenated portal vein via a 36 French cannula where a flow of 1 L/min was maintained at a pressure of 10 mmHg. The artery was connected with a 10 French cannula where a pulsatile flow (80/50 mmHg) was applied. The liver cleared lactate and produced bile with peak blood AST (2287 U/L) and ALT (1490 U/L) after 46 hours of perfusion. A custom perfusion loop was used (**Supplementary Figure S17**) and creatinine was measured in a Luer-to-Luer connector (**Supplementary Figure S18**) .

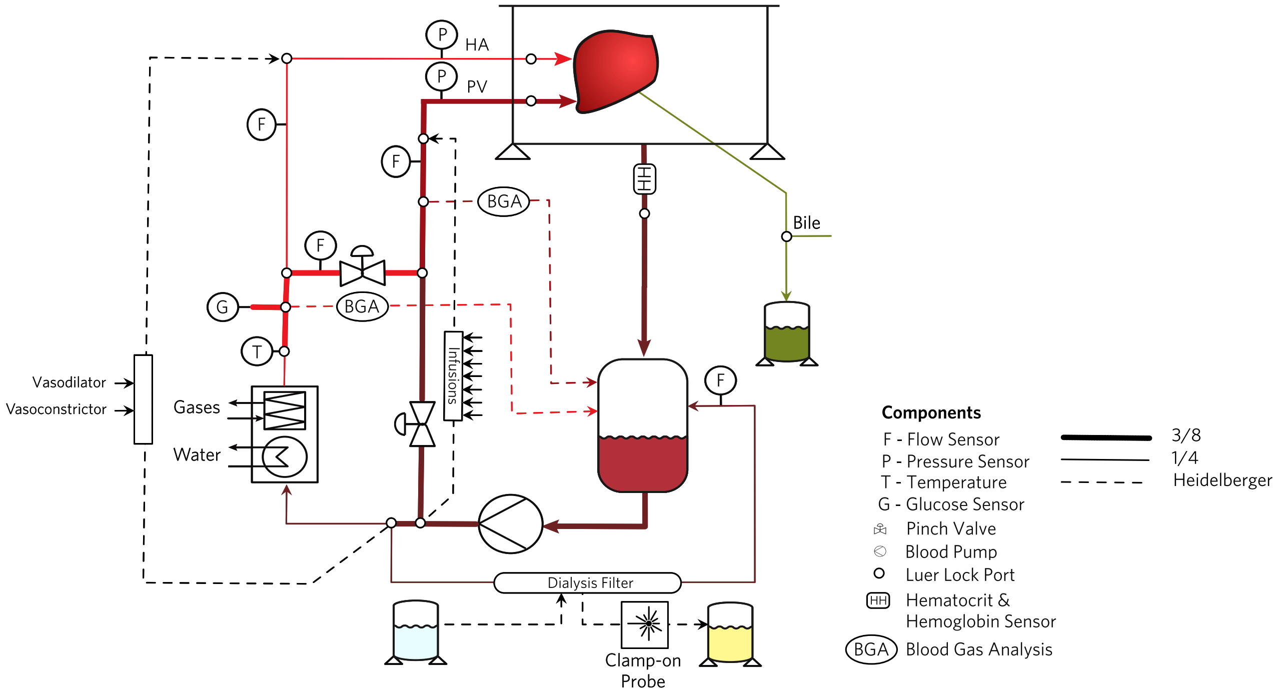

Supplementary Figure S17: Scheme of the perfusion loop for long term normothermic perfusion of a partial human liver

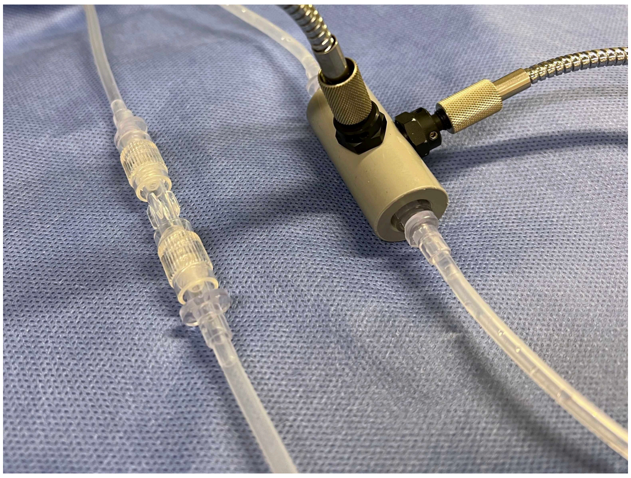

Supplementary Figure S18: Luer-to-Luer connector to measure creatinine in waste dialysate, once with clamp-on probe and once without

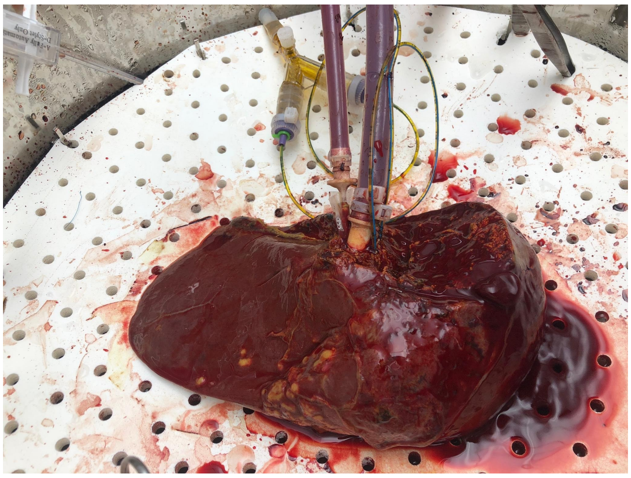

Supplementary Figure S19: Right hemi-liver during normothermic perfusion (vessels from left to right: bile ducts, hepatic artery, portal vein)

Experiments were approved by local authorities (Cantonal Ethics Committee Zurich KEK no. 2017-00412 and 2017-01292).

6 Normothermic Perfusion of Discarded Grafts

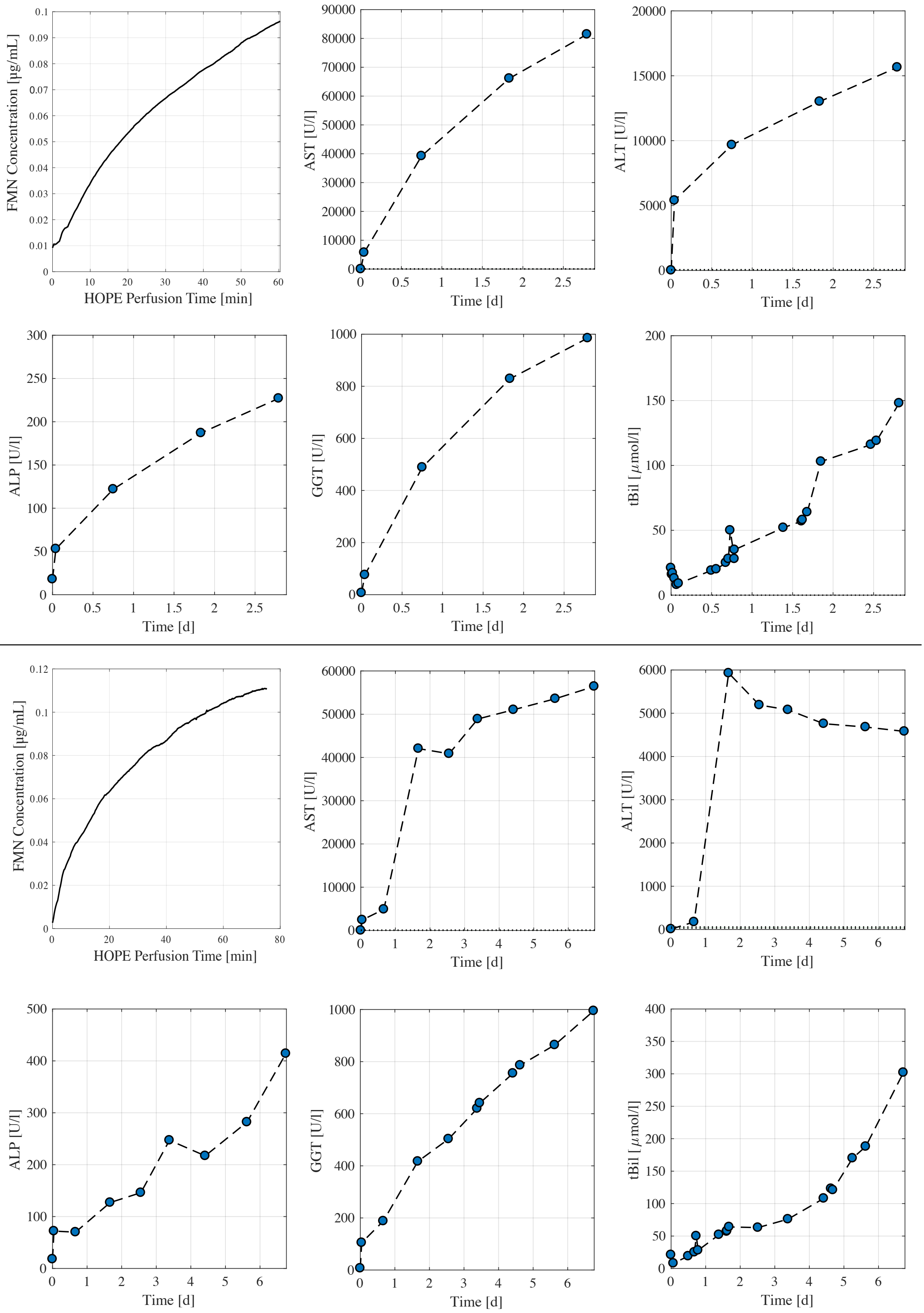

Supplementary Figure S20: Data of normothermic perfused discarded grafts

During the course of this study, two of the discarded grafts (Nr.13 and Nr. 23) were perfused on our custom normothermic perfusion system. Both grafts were discarded due to elevated FMN concentrations, measured with a plate reader. Real-time FMN data is shown in Supplementary Figure S20. Furthermore, AST, ALT, ALP, GGT and blood bilirubin concentrations are shown over the course of subsequent normothermic perfusion. Consistent increase of AST, ALT, ALP and GGT represents increasing liver inflammation while accumulation of perfusate bilirubin shows the inability of the liver to excrete bilirubin with bile. Taken together, we did not observe normal metabolic activity of these perfused grafts and interpret this as a signature of primary non-function during the normothermic perfusion, indicating poor graft quality and supporting the decision to have discarded the grafts.

7 FMN Measurement During Normothermic Perfusion

A 370 g resected liver was harvested from a female patient who suffered from an echinoccocus infection. Segments II, III, IVa and IVb were removed with cysts in segments III and IVb. Hemihepatectomy was performed in Zurich. It was subsequently cold-flushed and transported to our perfusion facility on ice. There it was connected to a normothermic perfusion system as described in[9–11]. 2.5 L of blood were circulated at a temperature of 37 °C. The liver was perfused for approximately 9 days. Online biomarker measurements were started with perfusion and stopped at the end of the experiment. Measurements were performed in waste dialysate by connecting a flow cell (Medica, M90392) to the waste dialysate tube. Measurements were conducted analogously to HOPE measurements. A separate calibration was performed to account for optical differences between Belzer MPS and dialysate. The flow rate of the waste dialysate was on average 250mL/h. While our experiment shows a proof of concept for measurements of FMN in waste dialysate, further measurements need to be validated with LC-MS and blood concentrations need to be compared to dialysate concentrations in detail.
Experiments were approved by local authorities (cantonal ethics commission KEK 2017–01292).

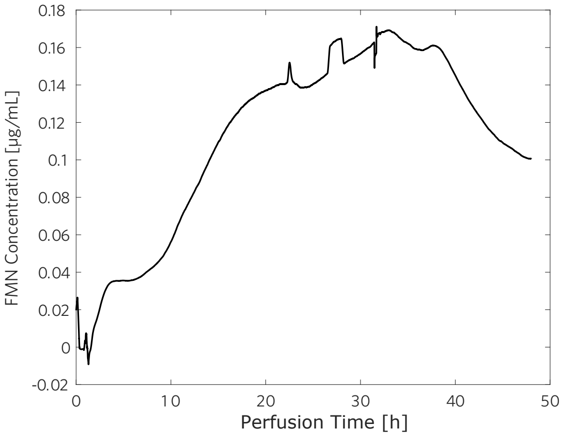

Supplementary Figure S21: FMN measurement during first 48 h of normothermic perfusion

References

1. Stepanova, A., Sosunov, S., Niatsetskaya, Z., Konrad, C., Starkov, A. A., Manfredi, G., Wittig, I., Ten, V. & Galkin, A. Redox-Dependent Loss of Flavin by Mitochondrial Complex i in Brain Ischemia/Reperfusion Injury. Antioxidants and Redox Signaling 31, 608–622. DOI: **10.1089/ars.2018.7693** (2019).
2. Zhang, J., Wang, Y. T., Miller, J. H., Day, M. M., Munger, J. C. & Brookes, P. S. Accumulation of Succinate in Cardiac Ischemia Primarily Occurs via Canonical Krebs Cycle Activity. Cell Reports 23, 2617–2628. DOI: **10.1016/j.celrep.2018.04.104** (2018).
3. Lotz, C., Herrmann, J., Notz, Q., Meybohm, P. & Kehl, F. Mitochondria and pharmacologic cardiac conditioning - at the heart of ischemic injury. International Journal of Molecular Sciences 22, 1–21. DOI: **10.3390/ijms22063224** (2021).
4. Hirst, J. Mitochondrial complex i. Annual Review of Biochemistry 82, 551–575. DOI: **10.1146/annurev-biochem-070511-103700** (2013).
5. Panconesi, R., Carvalho, M. F., Mueller, M., Meierhofer, D., Dutkowski, P., Muiesan, P. & Schlegel, A. Viability assessment in liver transplantation - what is the impact of dynamic organ preservation? Biomedicines 9, 1–25. DOI: **10.3390/biomedicines9020161** (2021).
6. Schaefer, P. M., Kalinina, S., Rueck, A., von Arnim, C. A. & von Einem, B. NADH Autofluorescence - A Marker on its Way to Boost Bioenergetic Research. Cytometry Part A 95, 34–46. DOI: **10.1002/cyto.a.23597** (2019).
7. Chouchani, E. T., Pell, V. R., Gaude, E., Aksentijevi , D., Sundier, S. Y., Robb, E. L., Logan, A., Nadtochiy, S. M., Ord, E. N., Smith, A. C., Eyassu, F., Shirley, R., Hu, C. H., Dare, A. J., James, A. M., Rogatti, S., Hartley, R. C., Eaton, S., Costa, A. S., Brookes, P. S., Davidson, S. M., Duchen, M. R., Saeb-Parsy, K., Shattock, M. J., Robinson, A. J., Work, L. M., Frezza, C., Krieg, T. & Murphy, M. P. Ischaemic accumulation of succinate controls reperfusion injury through mitochondrial ROS. Nature 515, 431–435. DOI: **10.1038/nature13909** (2014).
8. Schlegel, A., Muller, X., Mueller, M., Stepanova, A., Kron, P., de Rougemont, O., Muiesan, P., Clavien, P. A., Galkin, A., Meierhofer, D. & Dutkowski, P. Hypothermic oxygenated perfusion protects from mitochondrial injury before liver transplantation. EBioMedicine 60. DOI: **10.1016/j.ebiom.2020.103014** (2020).
9. Eshmuminov, D., Becker, D., Bautista Borrego, L., Hefti, M., Schuler, M. J., Hagedorn, C., Muller, X., Mueller, M., Onder, C., Graf, R., Weber, A., Dutkowski, P., Rudolf von Rohr, P. & Clavien, P.-A. An integrated perfusion machine preserves injured human livers for 1 week. Nature Biotechnology 38, 189–198. DOI: **10.1038/s41587-019-0374-x** (2020).
10. Clavien, P.-A., Dutkowski, P., Mueller, M., Eshmuminov, D., Bautista Borrego, L., Weber, A., Muellhaupt, B., Sousa Da Silva, R. X., Burg, B. R., Rudolf von Rohr, P., Schuler, M. J., Becker, D., Hefti, M. & Tibbitt, M. W. Transplantation of a human liver following 3 days of ex situ normothermic preservation. en. Nature Biotechnology. DOI: **10.1038/s41587-022-01354-7** (2022).
11. Mueller, M., Hefti, M., Eshmuminov, D., Schuler, M. J., Sousa Da Silva, R. X., Petrowsky, H., De Oliveira, M. L., Oberkofler, C. E., Hagedorn, C., Mancina, L., Weber, A., Burg, B., Tibbitt, M. W., Rudolf von Rohr, P., Dutkowski, P., Becker, D., Bautista Borrego, L. & Clavien, P.-A. Long-term Normothermic Machine Preservation of Partial Livers: First Experience With 21 Human Hemi-livers. en. Annals of Surgery 274, 836–842. DOI: **10.1097/SLA.0000000000005102** (2021).
